# Good practice in reaching and treating refugees in addiction care in Germany – a Delphi study

**DOI:** 10.1186/s12889-023-17446-1

**Published:** 2024-01-02

**Authors:** Panagiotis Stylianopoulos, Laura Hertner, Andreas Heinz, Ulrike Kluge, Ingo Schäfer, Simone Penka

**Affiliations:** 1 Department of Psychiatry and Neurosciences at the Charité Campus Mitte, Charité – Universitätsmedizin Berlin, Corporate Member of the Freie Universität Berlin and Humboldt-Universität Zu Berlin, Charitéplatz 1, 10117 Berlin, Germany; 2 Berlin Institute for Empirical Integration and Migration Research at the Humboldt Universität zu Berlin, Berlin, Germany; 3 Department of Psychiatry and Psychotherapy, University Medical Center Hamburg-Eppendorf, Hamburg, Germany; 4 Center for Interdisciplinary Addiction Research, University of Hamburg, Hamburg, Germany

**Keywords:** Addiction care, Refugees, Barriers, Access, Good practice, Drug use, Prevention

## Abstract

**Background:**

Health and adequate access to health care are human rights. Refugees are at risk for substance abuse. Despite the known structural and personal risk factors for abuse, refugees in Germany continue to face barriers to adequate addiction prevention and care, which is a violation of the fundamental human right to health care. The question arises as to how barriers for refugees in reaching addiction services and care can be overcome. In the presented study, strategies for good practices to deconstruct these barriers were identified.

**Method:**

A total of 21 experts participated in a three-round, consensus-oriented Delphi-Process. The experts represented five different fields: addiction care services, including specialized programs for women, refugee aid services, academia, policy-making and immigrants’ self-help services.

**Results:**

The Delphi-Process generated 39 strategies of good practice summarized in 9 major categories: Care System, Framework Conditions, Multilingualism, Information and Education, Access, Service-Level, Employee-Level, Employee-Attitudes and Networking.

**Conclusion:**

In order to guarantee human rights regarding health and adequate access to health care for refugees, institutional barriers limiting access to prevention and treatment programs for addictive disorders must be abolished. The identified good practice strategies for Germany, if widely implemented, could contribute to this aim. By opening up prevention and treatment facilities for refugees, other marginalized groups could also benefit. While some of the strategies need to be implemented at the institutional level, political steps are also required at the system level including, e.g. financing of adequate translation services.

**Supplementary Information:**

The online version contains supplementary material available at 10.1186/s12889-023-17446-1.

## Background

According to the United Nations High Commission for Refugees [1], the number of displaced people and refugees worldwide has reached an all-time high of 100 million people in 2022. In Germany, the total number of refugees and asylum seekers has increased over the last decade. In 2021, more than 1.9 million protection seekers,^1^ lived in Germany [2].

Studies show that a significant number of refugees (mis)use drugs [3–5]. Nonetheless, prevalence estimates are heterogeneous and inconsistent, partly because of different definitions of substance use and addiction [6].

Salas-Wright & Vaughn [7] found evidence that refugees in the US are less likely to meet substance use disorder criteria, especially if they were newly arrived refugees (less than 1 year), compared to non-refugee migrants or native-born Americans. The authors hypothesize these effects to be due to a high degree of legal protection, guidance and support refugees receive upon arrival (e.g. social welfare benefits or support provided by refugee aid organizations). Nevertheless, refugees in the post-migration setting are still subject to various socio-economic risk factors for substance (mis)use and addiction ranging from unemployment and poverty, marginalization and discrimination [8, 9], poor living conditions, lack of entitlement to work and family status [10], to coping with traumatic experiences both in the country of origin, as well as the host country [11, 12].

Health and adequate access to necessary health care is explicitly mentioned in both the constitution of the World Health Organization [13] and in Article 25 of the Universal Declaration of Human Rights [14]. All services should be accessible to all populations, especially to vulnerable populations such as refugees.

However, a systematically decreased utilization of addiction services [4, 15] and mental health care services in general [16–20] for immigrants and refugees is often indicated. While data focused on immigrants in general show an underrepresentation in addiction care, a closer look reveals a more heterogeneous picture, with immigrants using cocaine and opioids or seeking treatment for pathological gambling being overrepresented [15, 21]. There is no data available on refugees seeking help in addiction services in Germany, but some data suggest that first-generation immigrants – the definition of which includes refugees – use the addiction care system less than second-generation migrants [15].

Barriers to the addiction care system are diverse. For decades, immigrants and refugees themselves were considered responsible for barriers to the addiction care system [22]. These barriers were often attributed to socio-cultural barriers such as fear of stigma, lack of trust in health and social care professionals, as well as negative attitudes towards psychiatry and institutions [5, 23–25]. In recent years, deficits in the care system have increasingly been taken into account [26]. Factors that constitute access barriers on the part of care providers are uncertainty, irritation and triggering of fears and resentments due to unfamiliarity, fear of additional workload due to a particularly “difficult” and “burdened” group and seeking relief through delegation to special services. Furthermore, resistance due to fear of loss of competence as a result of culturalization is a factor. This can lead to over-interpreting a person’s supposed cultural background as a determinant and central explanation for behavior and attitudes, which results in not seeing them in all their multifaceted and intricate aspects; instead solely defining and reducing a person by their (presumed or real) cultural affiliation. This in turn requires special knowledge of the particular culture in order to work with the person [27]. Lack of provision of and funding for interpreting services alongside additional expected expenditure due to the need for interpreter services are often seen as a problematic issue with regard to access as well as care delivery [28–31]. Furthermore, perceived discrimination has also been described as a barrier to accessing services [32, 33]. Deimel [34] found that structural barriers limiting access to addiction care depend on residency status. Various barriers can impede access to healthcare, including unequal legal entitlements to care and administrative obstacles that make it challenging to obtain care even when there is a legal right to it. For example, separate insurance systems such as regular insurance cards or vouchers in German federal states, can add uncertainty over which institution will cover treatment costs [35], preventing refugees from gaining proper access.

In order to overcome the abovementioned barriers for immigrants and refugees when accessing (mental) health care in Germany, the multi-layered concept of “intercultural opening” is a guideline considered to be a standard for facilities for reaching and caring for immigrants [26, 36]. Additionally, several European good practice strategies have been identified to aim for reaching and serving immigrants and refugees equally. For instance, Priebe et al. [37] identified in a case vignette study various components of good practice to tackle the barriers and problems evolving among 16 European countries when it comes to delivering care for migrants. Specifically, in mental health services organizational flexibility with sufficient time and resources, working with relatives and social services, and good interpreting services were the three components of outstanding importance. Kluge et al. [38] followed up on these components and further examined the practical implementation of interpreting services in health care. The data revealed almost half of the services interviewed from all over Europe did not provide any type of interpreting services for patients with language barriers. The authors discuss that lack of payment regulations could be one of the reasons for the low provision and thus low utilization of interpreter services especially in countries where such services were not mainstreamed in the healthcare system.

Devillé et al. [39] found nine themes consisting of 186 factors representing strategies of good practice in health care in a Delphi-study across 16 EU member states. These included the establishment of low-threshold outreach programs, network activities within and outside of the care system, different forms of disseminating information about treatment and prevention within refugee communities as well as different caregivers, and most importantly, easy and equal access to all health care.

There has been little research regarding good practice, including the recommendations mentioned above, in helping refugees overcome barriers in addiction care. One qualitative study by McCleary et al. [40] showed three themes addressing barriers to successfully referring refugees to substance use treatment in the US. While the will for treating non-English speaking clients must be present, culture and language also have to be considered for successful referral and treatment of refugees. Clients’ preparation for substance use treatment can affect the success of referral and treatment utilization. Finally, coordination and support measures such as case management, alongside close accompaniment to and organization of appointments, can be necessary for successful referrals. However, previous studies of best practice strategies for reaching and serving refugees have not specifically addressed addiction services or focused on refugees as a distinct group outside the US.

The study presented in this paper used a three-round, consensus-oriented Delphi process aiming to produce priority good practice strategies for reaching and serving refugees in needs of addiction service, that can be implemented in Germany.

## Methods

The presented study was part of the PREPARE Consortium (“Prevention and treatment of substance use disorders in refugees”), funded by the German Federal Ministry for Education and Research.

**Fig. 1:**
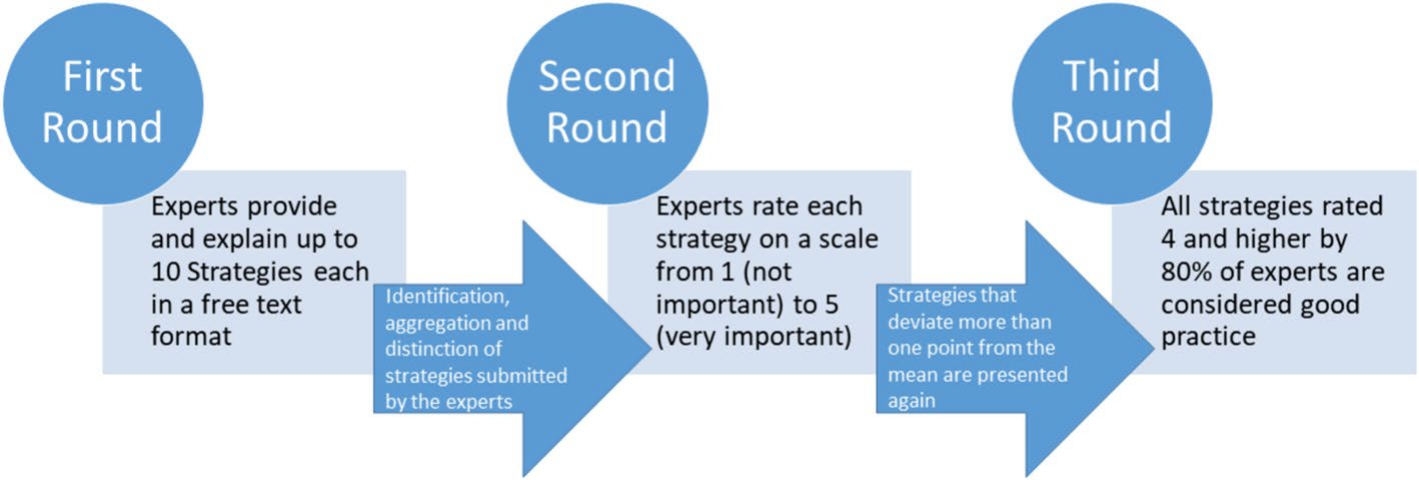
Step-by-Step overview on the expert-based three-round, consensus-oriented Delphi process conducted to identify strategies of good practice to reach and treat refugees in addiction care services

To identify good practice strategies for reaching and serving refugees in addiction services in Germany, we used an expert-based three-round, consensus-oriented Delphi process [41, 42]. For an overview of the procedure see Fig. 1. A similar study design was used by Devillé et al. [39] in order to identify strategies of good practice in health care. Differing from Devillé et al. [39] who used the Delphi survey to bring the group towards consensus on what constitutes good practice in health care for immigrants, the current study used it to identify priority good practices, in part based on experience, that target a specific sub-group of immigrants which are implementable in the context of Germany. Thus, analyzing reasons for non-consensus among the participants or between different stakeholders in the process of consensus-making were not the focus of the current Delphi study.

The Delphi method is an approach to collecting and creating expert opinions based on their expertise providing a level of consensus with a series of statements [43], that are provided to each expert anonymously in the subsequent round. The approach used involved a panel of experts presenting and rating strategies over three rounds. To achieve a high level of agreement, three rounds are viewed as sufficient [42, 44]. The research was conducted in German. The strategies and questionnaire (see Supplementary Material) were translated into English by two researchers and then reviewed by a native English speaker for the purpose of this publication and research dissemination.

### Panel formation

Our aim was to capture a heterogeneous representation of experts working in the fields of refugees and addiction, mental health, policy-making as well as key persons working with the target group. While the number of participants varies in Delphi-processes [42, 45], our panel consisted of 22 experts.

The main inclusion criteria for the panel were expertise related to refugees in general, in addiction services and support, or both. In order to ensure a broad representation of different viewpoints, the panel was aimed to consist of experts, half of whom have expertise in one of the different German fields of addiction support such as low-threshold street work, injection rooms, counseling centers, hospitals and rehabilitation centers as well as specialized programs for women. This ensured a wide array of views from within different addiction services. The other half included experts from the fields of refugee aid (e.g. employees in shelters), who often are the first to notice substance use among refugees and have experience on how referral to the care system can be managed; from academic researchers to complement knowledge from practice with meta-perspectives on public health approaches and marginalization of refugees; from policymakers to overcome structural barriers and realistic approaches; and from immigrants’ self-organization and addiction self-help (Narcotics Anonymous) to cover the perspective of the affected people themselves. The panelists were included because of their track record in the fields of refugee aid and or addiction support. Demographics of the panelists such as sociocultural or migrations status as well as education or profession were not recorded.

Identified experts were invited via an email that included a description of the study and an outline of the required contribution. After agreement to participate was gained, the questionnaire was sent via email together with a data protection information sheet and a consent form. Written informed consent was given by each expert before participation in the project. This project was granted human research ethics approval by the Charité – Universitätsmedizin ethics committee (number EA4/164/20).

A total of 51 experts were contacted overall, of whom 22 from across Germany agreed to share their experiences about strategies of good practice regarding the reach out to refugees and provision of addiction care services for them. Eleven had a background in addiction care services, including specialized programs for women, four were working in refugee aid services, two in academia, another two in the field of policy-making and three in immigrants’ self-help services. One expert had to be excluded after the first round due to incomplete answers.

### First round of Delphi-process

In the first round between September 2020 and October 2020, experts were asked to list, explain and justify in a free text format up to 10 good practice strategies in the field of addiction services for reaching refugees/asylum seekers and providing them with good care (see Fig. 1). Experts were asked to focus mainly on refugees that had arrived in 2015 or later and refugees of diverse gender and age. The question of what constitutes good practice was left to the experts. See supplementary material for the questionnaire.

A review of the collected strategies was subsequently conducted by a team of four researchers (PS, LH, SP, AM) experienced in qualitative data analysis. Strategies mentioned by the experts that included more than one strategy for reaching and caring for refugees in addiction services were divided into more selective and discrete strategies. Strategies mentioned by several experts were aggregated, even though different wording was used in reference to the same principles.

To identify, distinguish and aggregate all strategies mentioned, inductive coding with MaxQDA 2020 was performed. Two teams of two (PS & AM; LH & SP) researchers each analyzed all answers independently from each other. Each team was instructed to work as follows: firstly, the response of each expert was coded by two researchers independently. Discrepancies between those two coders were then discussed referring to each answering sheet and adjusted in the code system. Finally, the code systems from both teams were compared and integrated. As a result of this three-step process in-depth discussions and extensive re-considerations of the naming of strategies and grouping took place, resulting in a final code system. Each of the included codes, referred to a strategy for reaching and/or serving substance-using refugees. Descriptions of each strategy were derived from associated text sections.

The outcome of the first round of the Delphi process was 48 expert-based good practice strategies for reaching and treating refugees in addiction care. Each strategy included a title and a short explanatory text.

### Second round of Delphi-process

The second Delphi round took place from December 2020 to January 2021 as an online survey, hosted by LimeSurvey. Experts were asked to rate the importance of each strategy on a 5-Point-Likert scale (1 = not important, 5 = very important). No further explanation on why the Delphi-method required such ratings was provided. One expert was excluded because of no response in this stage of the process. Thereafter, the mean score was calculated based on the individual ratings of the remaining 21 experts for each strategy. See supplementary material for the questionnaire.

### Third round of Delphi-process

To reach a consensus among the experts interviewed within the third round, each expert was given feedback on their evaluation against the background of the group evaluation in an individual survey tool provided via email. If any expert’s rating differed by more than one point on the 5-Point-Likert scale from the group mean score of the respective strategy, they were asked to re-consider their rating. Each expert had the option to either adjust the individual rating to the displayed group mean, maintain the original rating, or increase the distance between the own rating of the strategy and the respective group mean. If an expert decided on one of the latter two options, a short explanation was required, which was then used to explain controversial strategies for which no consensus could be found. The Instructions were: “*This third round of the Delphi process aims for consensus among all experts interviewed regarding relevant strategies of “good practice“. At the same time, controversial strategies are identified for which no consensus can be found*.”

Finally, any strategy that was rated 4 or higher by at least 80% (*n* = 17) of the experts was considered good practice in terms of reaching and treating refugees in addiction services.

For better presentation, the consensus strategies were clustered into nine categories by the researchers after the third round. The categories are 1. Care System (three strategies), 2. Framework Conditions (two strategies), 3. Multilingualism (seven strategies), 4. Information and Education (three strategies), 5. Access (three strategies), 6. Service-Level (10 strategies), 7. Employee-Level (two strategies), 8. Employee-Attitudes (five strategies), 9. Networking (four strategies).

## Results

The three-round, consensus-oriented Delphi process identified 39 out of 48 initially proposed strategies as good practice for reaching and serving refugees in addiction services. Table 1 gives an overview of all strategies combined with the number of experts rating each strategy with at least 4 (important) and experts’ mean ratings in terms of importance in Delphi rounds two and three.

**Table 1:** Overview of all strategies

	Strategy	Second round ratings					M	Third round ratings					Number of Experts with 4 or 5 Rating	Category
		M	SD	Min	Max	Consensus		Mdn	SD	Min	Max	Consensus		
Good Practice Strategies	**1. Opening all existing addiction services to refugees**	**4.5**	**0.9**	**3**	**5**	**Yes**	**4.6**	**5**	**1**	**3**	**5**	**Yes**	**17**	**Care system**
	**4. Overcoming municipally-based support structures**	**4.2**	**0.9**	**2**	**5**	**Yes**	**4.4**	**4.5**	**1**	**3**	**5**	**Yes**	**19**	
	**6. Ensuring consistency of addiction support services**	**4.9**	**0.5**	**3**	**5**	**Yes**	**4.9**	**5**	**0**	**3**	**5**	**Yes**	**20**	
	**9. nationwide equal opportunities for refugees with regard to entitlement of benefits and right of use**	**4.7**	**0.6**	**3**	**5**	**Yes**	**4.8**	**4.5**	**0**	**4**	**5**	**Yes**	**21**	**Framework conditions**
	**10. Reduction of structural factors that facilitate or maintain addiction**	**4.7**	**0.6**	**3**	**5**	**Yes**	**4.8**	**4.5**	**0**	**4**	**5**	**Yes**	**21**	
	**11. Addressing people in their mother tongue as a gesture of welcome**	**4.2**	**0.8**	**3**	**5**	**Yes**	**4.3**	**5**	**1**	**3**	**5**	**Yes**	**18**	**Multilingualism**
	**12 Nationwide implementation of language mediation in addiction support facilities**	**4.5**	**0.7**	**3**	**5**	**Yes**	**4.6**	**4.5**	**1**	**4**	**5**	**Yes**	**21**	
	**13. Ensuring funding of language mediators**	**4.6**	**0.6**	**3**	**5**	**Yes**	**4.6**	**4.5**	**1**	**3**	**5**	**Yes**	**20**	
	**14 Fast and low-threshold availability of language mediators**	**4.5**	**0.8**	**3**	**5**	**Yes**	**4.7**	**4.5**	**1**	**3**	**5**	**Yes**	**20**	
	**15. Professionalism of the language mediators employed**	**4.6**	**0.6**	**3**	**5**	**Yes**	**4.6**	**4.5**	**1**	**3**	**5**	**Yes**	**20**	
	**16 Supervision for language mediators**	**4.3**	**0.7**	**3**	**5**	**Yes**	**4.4**	**4.5**	**1**	**3**	**5**	**Yes**	**20**	
	**17 Multilingualism of documents of the facility process**	**4.2**	**0.8**	**3**	**5**	**Yes**	**4.4**	**4.5**	**1**	**3**	**5**	**Yes**	**19**	
	**18. Provision of centrally designed, multilingual information on substances, substance use and addiction**	**4.3**	**0.9**	**2**	**5**	**Yes**	**4.3**	**4.5**	**1**	**2**	**5**	**Yes**	**18**	**Information and Education**
	**19. Passing on (bundled or centrally designed) multilingual information on addiction-related care services and framework conditions**	**4.3**	**0.9**	**2**	**5**	**Yes**	**4.4**	**4.5**	**1**	**2**	**5**	**Yes**	**19**	
	**20. Outreach information work in the living environment of refugees.**	**4.4**	**0.9**	**2**	**5**	**Yes**	**4.5**	**4.5**	**1**	**3**	**5**	**Yes**	**19**	
	**21. Raising awareness of addiction issues among those involved in refugee assistance**	**4.6**	**0.7**	**3**	**5**	**Yes**	**4.6**	**4.5**	**1**	**3**	**5**	**Yes**	**20**	**Access**
	**22. Use of key persons as door openers**	**4.3**	**0.9**	**2**	**5**	**Yes**	**4.4**	**4.5**	**1**	**2**	**5**	**Yes**	**20**	
	**24. Qualification of and work with “bridge builders**	**4.1**	**0.8**	**3**	**5**	**Yes**	**4.3**	**4**	**1**	**3**	**5**	**Yes**	**19**	
	**25. Ensuring low-threshold access to addiction support services**	**4.6**	**0.7**	**2**	**5**	**Yes**	**4.6**	**5**	**1**	**2**	**5**	**Yes**	**20**	**Service-Level**
	**26. Emphasis on discretion and anonymity**	**4.7**	**0.6**	**3**	**5**	**Yes**	**4.7**	**4.5**	**1**	**3**	**5**	**Yes**	**20**	
	**27. Ensuring participation and active involvement of people affected by addiction in the process of developing services and materials**	**4.2**	**0.9**	**2**	**5**	**Yes**	**4.3**	**4.5**	**1**	**2**	**5**	**Yes**	**19**	
	**28. Participation of refugees in self-help activities**	**4.3**	**0.9**	**3**	**5**	**Yes**	**4.5**	**4.5**	**1**	**3**	**5**	**Yes**	**20**	
	**29. Early intervention for substance use among refugees**	**4.3**	**1**	**2**	**5**	**Yes**	**4.4**	**4**	**1**	**2**	**5**	**Yes**	**17**	
	**31. Outreach counseling in the immediate surroundings of refugees**	**4.3**	**0.8**	**2**	**5**	**Yes**	**4.4**	**4.5**	**1**	**2**	**5**	**Yes**	**20**	
	**32. Outreach work in places where drugs are consumed**	**4.3**	**1**	**2**	**5**	**Yes**	**4.5**	**5**	**1**	**3**	**5**	**Yes**	**19**	
	**33. Outreach work to build up relationships**	**4.4**	**0.9**	**2**	**5**	**Yes**	**4.6**	**5**	**1**	**3**	**5**	**Yes**	**19**	
	**34. Regularity and durability in the relational work with refugees**	**4.3**	**0.9**	**2**	**5**	**Yes**	**4.4**	**4.5**	**1**	**2**	**5**	**Yes**	**19**	
	**35. Accompanying clients**	**4.3**	**0.7**	**3**	**5**	**Yes**	**4.3**	**5**	**1**	**3**	**5**	**Yes**	**19**	
	**36. Trainings for addiction support professionals that addresses the living situation of refugees**	**4.6**	**0.6**	**3**	**5**	**Yes**	**4.7**	**4.5**	**0**	**4**	**5**	**Yes**	**21**	**Employe-Level**
	**38 Promoting diversity in teams**	**4.5**	**0.8**	**3**	**5**	**Yes**	**4.6**	**4.5**	**1**	**3**	**5**	**Yes**	**20**	
	**39. Understanding and acceptance of substance use as a coping strategy**	**4.6**	**0.7**	**3**	**5**	**Yes**	**4.7**	**5**	**0**	**4**	**5**	**Yes**	**21**	**Employe-Attitudes**
	**40 Adopting an appreciative, living environment-oriented attitude**	**4.6**	**0.7**	**3**	**5**	**Yes**	**4.7**	**4.5**	**1**	**3**	**5**	**Yes**	**20**	
	**41 Cross- and transcultural competences in attitude and reflection**	**4.5**	**0.8**	**2**	**5**	**Yes**	**4.6**	**4.5**	**1**	**3**	**5**	**yes**	**20**	
	**42. Adopting a gender-sensitive attitude**	**4.2**	**1.1**	**1**	**5**	**No**	**4.3**	**4.5**	**1**	**1**	**5**	**No**	**18**	
	**43 Coping mechanisms and setting boundaries as a competence of professionals**	**4.1**	**0.9**	**3**	**5**	**Yes**	**4.2**	**4.5**	**1**	**0**	**5**	**Yes**	**17**	
	**44. Networking of all stakeholders involved in the care of drug users**	**4.5**	**0.6**	**3**	**5**	**Yes**	**4.5**	**5**	**1**	**3**	**5**	**Yes**	**20**	**Networking**
	**45. Multidisciplinary networking beyond addiction support services**	**4.3**	**0.9**	**2**	**5**	**Yes**	**4.3**	**4.5**	**1**	**2**	**5**	**Yes**	**18**	
	**47. Considering networking financially and conceptually**	**4.1**	**0.9**	**2**	**5**	**Yes**	**4.2**	**4.5**	**1**	**2**	**5**	**Yes**	**18**	
	**48. Establishment of in-depth inter-institutional cooperation**	**4.1**	**0.7**	**3**	**5**	**Yes**	**4.3**	**4.5**	**1**	**3**	**5**	**Yes**	**20**	
**Strategies not considered good practice**	**2. Creation of specific services for refugees**	**4.2**	**1**	**2**	**5**	**No**	**4.3**	**5**	**1**	**3**	**5**	**Yes**	**16**	**Strategies without consensus**
	**3. Establishment of services for individual subgroups of refugees**	**3.9**	**0.9**	**2**	**5**	**Yes**	**4**	**4.5**	**1**	**3**	**5**	**Yes**	**15**	
	**5.Enabling flexible project development and implementation**	**4**	**1.1**	**1**	**5**	**No**	**4.1**	**4.5**	**1**	**3**	**5**	**Yes**	**15**	
	**7. Management of requirements planning by policy-makers (federal government, state governments and municipalities)**	**3.4**	**1**	**2**	**5**	**Yes**	**3.6**	**4.5**	**1**	**2**	**5**	**Yes**	**10**	
	**8. Integration of science and practice**	**3.8**	**1**	**1**	**5**	**No**	**3.7**	**4**	**1**	**1**	**5**	**Yes**	**13**	
	**23. Working with relatives**	**4**	**0.9**	**2**	**5**	**Yes**	**4**	**4**	**1**	**2**	**5**	**Yes**	**15**	
	**30. Creating and maintaining a welcoming environment across all services**	**3.7**	**1.2**	**1**	**5**	**No**	**3.5**	**4.5**	**1**	**1**	**5**	**No**	**12**	
	**37. Cultural sensitivity of professionals [1]**	**3.8**	**1.3**	**1**	**5**	**No**	**3.7**	**4**	**1**	**1**	**5**	**No**	**13**	
	**46. Networking with civil society stakeholders**	**4**	**0.8**	**3**	**5**	**Yes**	**4**	**4.5**	**1**	**3**	**5**	**Yes**	**14**	

[1] Cultural sensitivity as it is used here does refer to a static understanding of culture. It draws back to the need of knowledge about norms, values and attitudes of specific cultures while dismissing heterogeneity and transitions within and between cultures and individuals [46]

### Care system

The strategies of this category aimed to decrease structural barriers that are not necessarily refugee-specific, and to call for an opening of already existing programs for refugees. The category includes the strategy: *“Opening all existing addiction services to refugees” (M = 4.57; SD = 0.81; Mdn = 5)* in order to ensure seamless, continuous support, care and treatment close to home and in line with needs in all areas of addiction service. Taking into account the mobility of some refugees (e.g. their registered address differs from their place of residence), the identified good practice strategy “Overcoming municipality-based support structures” (*M = 4.38; SD = 0.67; Mdn = 4,5*) could enable continuity in care (despite e.g. frequent changes of accommodation). *“Ensuring consistency of addiction support services” (M = 4.68; SD = 0.48; Mdn = 5)* as a good practice strategy that also met the consensus threshold addresses the need for stable, sufficient and permanent funding of addiction support services*.*

### Framework conditions

Strategies of this category aimed for the meta-level, such as improvement of the conditions of refugees’ everyday lives and their entitlement to use (mental) health care services. The initially suggested strategies: “*Nationwide equal opportunities for refugees with regard to entitlement of benefits and right of use” (M = 4.76; SD = 0.44; Mdn = 4,5)* and “*Reduction of structural factors that facilitate or maintain addiction” (M = 4.76; SD = 0.44; Mdn = 4,5)* were considered relevant good practice by the experts and met the consensus threshold. The latter strategy refers to structural aspects in the living situation of refugees, such as lengthy asylum procedures, accommodation, difficult access to training and work etc., that facilitate addiction and suggests them to be taken seriously and reduced or eliminated in order to ensure social participation regardless of the legal residence status*.*

### Multilingualism

Strategies of this category focused on ensuring clear linguistic communication and understanding for two purposes; to ensure the transmission of correct information and as a signal of openness and welcome. All but the last of these strategies dealt with the implementation of and access to language mediation as well as language mediators and their professionalism. All of the seven originally identified strategies were considered good practice and met the consensus threshold among the experts involved, and included: *“Addressing people in their mother tongue as a gesture of welcome” (M = 4.29; SD = 0.72; Mdn = 5), “Nationwide implementation of language mediation in addiction support facilities” (M = 4.62; SD = 0.50; Mdn = 4,5)* in order to ensure that all refugees can make use of regular care services and thus enable, for example, further referral in the context of addiction counseling or follow-up treatment after clinical withdrawal and*,* “E*nsuring funding of language mediators” (M = 4.57; SD = 0.60; Mdn = 4,5)* were among these strategies. Additionally*,* “*Fast and low-threshold availability of language mediators” (M = 4.67; SD = 0.58; Mdn = 4,5)* e.g. via a pool of language mediators - whether for face-to-face, telephone or video mediation, is among the set of consensual and relevant good practice strategies in the category multilingualism. Furthermore, the category includes the strategies*,* “*Professionalism of the language mediators employed” (M = 4.62; SD = 0.59; Mdn = 4,5)* including for instance the correct translation without personal judgement*, “Supervision for language mediators” (M = 4.43; SD = 0.60; Mdn = 4,5) and “Multilingualism of documents of the facility process” (M = 4.38; SD = 0.67; Mdn = 4,5),* e.g. data protection, treatment and confidentiality agreements*.*

### Information and education

Strategies of this category dealt with the way information about the addiction support system and addiction should be designed, presented and passed on. All of the originally identified strategies met the consensus threshold and were considered relevant good practice: “*Provision of centrally designed, multilingual information on substances, substance use and addiction” (M = 4.33; SD = 0.86; Mdn = 4,5)* includes the means of centrally designed flyers, films, apps, social media, contributions in non-native German-language television programs or broadcasts to establish an understanding of addiction as a treatable health problem/illness.*,* “*Passing on (bundled or centrally designed) multilingual information on addiction-related care services and framework conditions” (M = 4.38; SD = 0.80; Mdn = 4,5)* addresses the need to publicize diverse addiction care services as well as their range of services and essential framework conditions (such as confidentiality, data protection, anonymity, addiction support as non-governmental) publicized among refugees. Last but not least*, “Outreach information work in the living environment of refugees” (M = 4.48; SD = 0.68; Mdn = 4,5),* e.g. in German/integration courses, refugee accommodation, self-help groups, neighborhood centers, social media, serve the means of prevention and education*.*

### Access

Strategies of this category referred to access to refugee populations and how refugees could be reached by addiction support services. The named strategies focused on educating multipliers within the refugee support services as well as key persons or “bridge builders” (peers) from within the community on topics of addiction, addiction care and substance (mis)use. Three strategies were deemed consensual and relevant good practice among the experts: “*Raising awareness of addiction issues among those involved in refugee assistance” (M = 4.62; SD = 0.59; Mdn = 4,5)*, e.g. through training courses, ensures professionals and volunteers working with refugees to be informed about substance use and addiction support services and their insecurities in dealing with substances and addiction to be reduced. The strategy *“Use of key persons as door openers* “*(M=4.38; SD=0.74; Mdn=4,5)* addresses the relevance to making parents, mothers, caregivers, stakeholders of a community or others who are in good contact with refugees multipliers and open the doors for addiction support services to approach the target groups. Finally, “*Qualification of and work with “bridge builders” (M = 4.29; SD = 0.64; Mdn = 4)* describes how to integrate peers, buddies, health or integration facilitators, lay helpers, etc. actively into the addiction support work as qualified bridge builders to establish contact and trust with refugees and clarify (culturally conditioned) misunderstandings. Aspects such as financial remuneration, continuous qualification, connection and supervision are incorporated into this good practice strategy*.*

### Service-level

In general, strategies of this category aimed to reduce barriers for refugees within existing services, remove inhibitions, strengthen trust and empower refugees The following 10 strategies were deemed consensual and relevant strategies of good practice among the experts: “*Ensuring low-threshold access to addiction support services” (M = 4.62; SD = 0.74; Mdn = 5),* e.g. through the establishment of telephone services in different languages, open regular counseling services (also for relatives), services without a prior appointment (e.g. in shelters); *“Emphasis on discretion and anonymity” (M = 4.67; SD = 0.58; Mdn = 4,5), “Ensuring participation and active involvement of people affected by addiction in the process of developing services and materials” (M = 4.33; SD = 0.80; Mdn = 4,5), “Participation of refugees in self-help activities” (M = 4.52; SD = 0.60; Mdn = 4,5), “Early intervention for substance use among refugees” (M = 4.38; SD = 0.92; Mdn = 4). Furthermore, “Outreach counseling in the immediate surroundings of refugees” (M = 4.38; SD = 0.74; Mdn = 4,5),* e.g. in the form of regular open office hours in a shelter, in German/integration courses, self-help groups, neighborhood centers or social media*, “Outreach work in places where drugs are consumed” (M = 4.48; SD = 0.68; Mdn = 5)* establishing contact as well with refugee users *and “Outreach work to build up relationships” (M = 4.57; SD = 0.68; Mdn = 5)* by addiction support workers being regularly present in the (living) environment of refugees and focusing their conversations on topics relevant to the person and not on the care mandate of addiction support services are among the set of good practice strategies in the category service-level. Aiming for a trustful relationship with clients, *“Regularity and durability in the relational work with refugees” (M = 4.38; SD = 0.80; Mdn = 4,5)* acknowledges as a strategy the time investment required and suggests fixed contact persons, e.g. the same staff member, the same language mediator. Last but not least, *“Accompanying clients” (M = 4.33; SD = 0.66; Mdn = 5),* e.g. *to* authorities, day-structuring services, counseling centers, and clinics, is suggested to stabilize the situation and facilitate the connection to high-threshold services (e.g. substitution)*.*

### Employee-level

Strategies of this category aimed to promote diversity in terms of migration history and/or non-German (native) language skills in employee teams, and to address the acquisition and extension of knowledge of existing refugee-specific legal frameworks. The category includes two strategies that met the consensus threshold and were considered relevant good practice: *“Promoting diversity in teams” (M = 4.67; SD = 0.48; Mdn = 4,5)* aiming for partially overcoming language barriers, but also to change the discourse in facilities and *“Trainings for addiction support professionals that addresses the living situation of refugees* and *Promoting diversity in teams” (M = 4.57; SD = 0.60; Mdn = 4,5)*. The latter strategy addresses the need for addiction support professionals to receive specialized training regarding the complex socio-political conditions and living situations of refugees, e.g. aspects of asylum and residence law, family reunification, regulations on coverage of costs and the responsibilities of cost bearers, refugee assistance services, employment opportunities, etc. They should be informed and made aware of these issues, but they do not take over tasks from other areas of work (such as (asylum) legal counseling centers).

### Employee-attitudes

Strategies of this category focused on the awareness of addiction service employees towards concepts that seem foreign, or contradict one’s own convictions, and on the development of a professional attitude that involved focusing on each client as a unique case. The strategies demonstrate the importance of continuous self-reflection, encouraging individuals to reflect on their own and others’ conclusions, assumptions, and prejudices, fostering a deeper understanding and awareness. *“Understanding and acceptance of substance use as a coping strategy” (M = 4.71; SD = 0.46; Mdn = 5), “Adopting an appreciative, living environment-oriented attitude” (M = 4.67; SD = 0.58; Mdn = 4,5), “Cross- and transcultural competencies in attitude and reflection” (M = 4.62; SD = 0.59; Mdn = 4,5), “Adopting a gender-sensitive attitude” (M = 4.29; SD = 1.01; Mdn = 4,5),* and *“Coping mechanisms and setting boundaries as a competence of professionals” (M = 4.24; SD = 0.77; Mdn = 4,5).* As for cross- and transcultural competencies the following were suggested: meeting refugee clients with an open, curious and questioning attitude at eye level instead of with prejudice and judgement; shifting the focus away from the “culture” of the other person as conceived by the nation-state or cultural circles but on one’s own attitude; and the ability to reflect on one’s own, often Eurocentric understanding of health and illness are outlined.

### Networking

Finally, strategies clustered in the category networking outlined the importance of networking on different levels. Networks are established in order to spread information about services, but also to further establish cooperation e.g. common action guidelines. Networking is time-consuming and should thus be considered as a specific target when conceptualizing a service. Four strategies were consensually deemed relevant good practice: “*Networking of all stakeholders involved in the care of drug users” (M = 4.52; SD = 0.60; Mdn = 5)* includes a regular exchange of information and support among addiction support professionals. *“Multidisciplinary networking beyond addiction support services” (M = 4.29; SD = 0.85; Mdn = 4,5)* goes beyond the field of specialized addiction support services, and involves: e.g. migration counseling, family counseling, help for the homeless, and/or health care, into the networking efforts. This aims, amongst others, at an interdisciplinary exchange of expertise and referrals being made in line with the client’s needs. These strategies are rated just as important as *“Considering networking financially and conceptually” (M = 4.19; SD = 0.81; Mdn = 4,5)* and the *“Establishment of in-depth inter-institutional cooperation” (M = 4.29; SD = 0.56; Mdn = 4,5)*.

### Strategies without consensus

Nine strategies did not reach consensus. More than half (*n* = 5) were those grouped under the first category of the “care system” and included: “*Creation of specific services for refugees*” (*M = 4.33; SD = 0.86; Mdn = 5*) to meet their cultural, linguistic and trauma-specific needs; “*Establishment of services for individual subgroups of refugees*” (*M = 3.95; SD = 0.74; Mdn = 4,5*); “*Enabling flexible project development and implementation*” (*M = 4.05; SD = 0.80; Mdn = 4,5*), e.g. to adapt projects to unforeseen needs; “*Management of requirements planning by policy-makers (federal government, state governments and municipalities)*” (*M = 3.57; SD = 0.81; Mdn = 4,5*) in order to prepare addiction support facilities for the expected number of refugees; and “*Integration of science and practice*” (*M = 3.67; SD = 0.91; Mdn = 4*) to ensure evidence-base of the interventions.

The strategies “*Working with relatives*” (*M = 4.68; SD = 0.48; Mdn = 4*) under the category “Access” and “*Creating and maintaining a welcoming environment across all services*” (*M = 3.52; SD = 1.03; Mdn = 4,5*) under the category “Service level” also did not reach the consensual threshold among the experts.

“*Cultural sensitivity of professionals*^2^” (*M = 3.67; SD = 1.20; Mdn = 4*) was the only item under the category “Employment level” that did not meet consensus. The strategy describes the ability of professionals to act in a culturally sensitive manner when dealing with refugees. The prerequisite for this ability was listed as knowledge about the countries of origin and cultural backgrounds (politics, society and culture), cultural characteristics, religions, value systems and world views of refugees, as well as culture-specific characteristics of addiction and mental illness.

Finally, the strategy “*Networking with civil society stakeholders*” (*M = 4; SD = 0.84; Mdn = 4,5*) under the category “Networking” did not reach consensus and thus was excluded from the final list. This strategy describes the extra efforts made by the professionals in addiction care to reach out to civil society organizations and stakeholders (e.g. churches, sports clubs, and language schools) to refer their clients to social and leisure activities as an alternative strategy to substance use in a way that meets their needs.

## Discussion

A total of 39 priority strategies of good practice in reaching and treating refugees were identified in our three round consensus oriented Delphi-process, with 21 experts working in the fields of refugees and addiction, mental health, policy-making and key persons working with the target group.

This study confirms and extends the results of Devillé et al. [39] and Priebe et al. [47]. Our study results also highlight strategies focusing on equal access to health care, low threshold outreach programs both for engagement with and treatment of the target group, networking or collaboration between different services both within and without the specific care system, and provision of information about services and substances as a means of empowering those affected to act in a self-determined manner.

A major difference was the additional relevance of reducing structural, addiction-promoting circumstances refugees are exposed to on a strategy level, which became clear in the context of the strategy “*Reduction of structural factors that facilitate or maintain addiction”*. While Priebe et al. [37] also found that social issues such as housing problems were considered as problem areas by the respondents they were not reflected in the good practice strategies. Post-migration stressors, especially in the context of the living situation of refugees, but also legal status concerning prospects and possibilities of e.g. work and meaningful activities, facilitate addiction. Since modifying socio-political structures that contribute to addiction require political change and commitment at a higher level, it remains to be seen how individual addiction care facilities can make an impact. Nevertheless, building on previous studies [10, 48] our current study contributes to wider policymaking by not only addressing the importance of the socio-ecological model of prevention which is beyond the conventional model of prevention focused on individual behavior, but also by identifying it as one of concrete good practice strategies to be implemented.

To enable successful treatment, structural factors embedded in refugees’ social ecology, such as formal access to the job market, family reunification, and residence prospects, have to be addressed [10, 49]. Socio-political solutions to combat marginalization are needed. Another difference in comparison to other study results [39, 47] is that our results highlighted the need to open up existing services to refugees rather than creating separate special projects and services for refugees, which is the common procedure in Germany. This is especially illustrated by the strategy “*Opening all existing addiction services to refugees”.* Strategies such as “*Ensuring funding of language mediators*” aim to enable the opening of existing regular services. The explicit rejection of “special projects” for refugees was also confirmed by the failure to reach consensus as good practice strategies of the initially presented strategies “*Creation of specific services for refugees”*. Strategies aiming at special projects for refugees promote “othering” defined by Weis [50] as a “process that serves to mark and name those who are thought to be different from oneself”. It is a means of securing and defining one’s identity through stigmatization and distancing of others in an “us-them” separation, which leads to exclusion and has hence been identified as a barrier to access and delivery of health services [51]. Contrasting our study results and those by Devillé et al. [39], language mediation was not part of the best practice strategies described by Priebe et al. [47]. This could be due to the fact that these authors did not focus exclusively on refugees/asylum seekers. The results of Deville et al. [39] and our own study identified different aspects of language mediation and multilingual material as good practice for better communication and proper treatment. In order to do justice to the great linguistic diversity of refugees – for instance, refugees from 152 countries applied for asylum in Germany in 2022 [52] – the use of language mediation is essential and bilingual staff cannot cover this diversity.

In Germany, funding of language mediators in health care is often a decision made by health institutions based on their stance towards opening access to non-German speakers by allocating funds [35], and not an entitlement through e.g. health insurance. Straßmayr et al. [24] point out that some care providers have implemented strategies concerning access to help or language mediation either despite (and sometimes against) current legislation, which in turn places care providers in either an ethical or a financial dilemma. This shows a need for change on a policy level to provide legislation that tackles socio-political barriers to accessing healthcare [53], a need that the current coalition government of the Federal Republic of Germany addresses in the 2021 coalition agreement [54].

While “Working with relatives” was identified as a good practice by Priebe et al. [47], this item did not reach consensus in our current study. An explanation for this could be our study’s specific focus on refugees and addiction care while Priebe et al. study included wider migrant populations in the broader context of healthcare [47]. Family separation and lack of family support for single travelers have been identified as post-migration stressors contributing to mental health issues and increased risk for addiction [5, 10]. In Germany, 61% of all refugees are male [55] of whom 63.4% are between the age of 18 and 65 living without a spouse or relative in their household [56]. Thus, the limited possibility to work with relatives in the context of addiction care for refugees in Germany could have influenced some experts’ decision not to rate this item as important as others.

Networking and institutional cooperation with relevant stakeholders were considered important by the experts in our study with four specific network-related strategies included in the final list. However, “*networking with civil society stakeholders*” such as churches and sports clubs did not achieve consensus. This may seem contradictory given that the strategy “*Qualification of and work with ‘bridge builders*’” (under the category “Access”), which include community and civil society actors, did meet consensus. An explanation for this could be that networking with civil society actors who do not specifically work with migrants in need of addiction care might have been considered too vague as a strategic choice. A peer approach in which a common language facilitates sense of belonging is known to help alleviate initial inhibitions of addiction care and/or help initiate a conversation [57]. Thus, such targeted networking or working with specialized civil or community actors were likely preferred to working with civil society stakeholders working with wider general populations.

*“Cultural sensitivity of professionals”* as it was described as an initially proposed strategy, refers to the need of knowledge about cultural background, culture, value systems and world views of refugees. This does not only build on an static and definite understanding of culture [46], but further contributes to undesirable dynamics of othering. Weis [50] describes othering as the process that “serves to mark and name those thought to be different from oneself”, consequently making it difficult to work with immigrants without sufficient knowledge. According to Akbulut and Razum [58], in the context of public health, the concept of “Othering” sheds light on the relationship between minority status and health disparities. Othering pertains to individuals depicted as the opposite of and in contrast to the “We,” and their exclusion allows the “We” to shape its identity. This contrast can help to comprehend group-related differences in health outcomes, as well as inadequate utilization and/or provision of certain health services due to consequences of othering on an individual, institutional and contextual level. Following this strain of thought, the initially proposed strategy of *“Creation of specific services for refugees”* constitutes othering.

In contrast, “*Cross- and transcultural competencies in attitude and reflection”* gained consensus *and* was considered good practice by the expert panel. The main difference between these strategies is the underlying concept of culture/ cultural difference and background. Prevailing paradigms of culture do not consider it as a static and distinct entity, but as complex, dynamic, and hybrid [46]. Cultural complexity denotes the existence of intricate dynamics, diverse perspectives, and ongoing negotiations within a specific culture. Cultural dynamics and hybridity encompass the temporal and historical aspects of cultural processes, emphasizing their ever-changing, interacting, and interdependent nature. Similarly, Steinhäuser et al. [59] found in a literature review two contrasting concepts of culture as static and dynamic. The static concept views culture as homogenous unit often used interchangeably with country of origin or ethnicity, whereas the dynamic concept of culture considers culture as an individually self-produced hybrid belief system that emerges in a procedural manner and is continuously changeable. Within the latter, geographical experiences, external living conditions, and socio-cultural backgrounds influence the construction of every person’s environment. Applied to the strategies of *“Cultural sensitivity of professionals”* and “*Cross- and transcultural competencies in attitude and reflection”,* the former requires knowledge of a multitude of backgrounds and origins, which in turn, assumes a static understanding of culture that encompasses all members of a specific cultural background. The latter refers to open-minded curiosity towards other people as well as a self-reflective process, especially in light of one’s own background in the sense of a non-discriminatory attitude. This is, according to Steinhäuser et al. [60], a protracted active process of self-reflection while working with immigrants. Transcultural competence could also be perceived as more pragmatic opposed to accumulation of cultural knowledge. However, if this is internalized, it immanently leads to a change of attitude or a more dynamic concept of culture. The strategies “*Creation of specific services for refugees”, “Establishment of services for individual subgroups of refugees*” and “*Cultural sensitivity of professionals”,* which were not ultimately determined to be good practice, refer to a static understanding of culture. The experts we interviewed thus advocated for an open concept of culture.

To our knowledge, this is the first study on good practice strategies in reaching and treating refugees in addiction care in Germany. The findings represent knowledge gathered from different experts from a multitude of fields. By expanding the selection process to experts from fields beyond addiction services, representativeness was addressed as much as possible. However, the aim of a multi-stakeholder Delhi study is to represent variety of perspectives rather than aiming for “full representativeness” of expert views [42]. In future research, a participatory approach with people who are users of the care system should be conducted. The study was conducted in and about Germany; an application to a broader context is, therefore, limited. However, the results are similar to previous internationally conducted studies [39, 47]. While these studies had a broader range of marginalized groups and broader fields of application, the similarities of the results might point at a need to generally aim for inclusive health and addiction care services. Eventually, implementing the strategies of good practice presented serves to reduce genuine barriers to adequate addiction care service provision and utilization, and thus might be beneficial not only for refugees, but also for other marginalized groups.

As part of a different work package of the PREPARE research and intervention consortium, a study on the status of implementation of the identified good practice strategies in the German addiction services was conducted in 2022 and will be published in the future. Additionally, a list of the consensus on good practice strategies has been published in German in order to facilitate the application [61].

## Conclusion

The results of this study confirm the available results regarding good practice for marginalized population groups in Europe, most of which stem from studies with small sample sizes. Contextualizing them for the German addiction care system adds to the growing body of work showing the need to open up (health)services to refugees. The process of opening up institutions for refugees cannot be successful as long as policy frameworks concerning marginalization are not adequate.

More research on the implementation of these strategies is necessary to validate our findings. Furthermore, reducing barriers and opening up existing services is not adequate as long as structures such as funding for language mediation are not made available. Therefore, on a political level, legislation needs to be adjusted in order to facilitate lasting change, not only in terms of access to health care but also in terms of health prevention by addressing structural factors that impair equal access to addiction prevention and treatment.

### Supplementary Information

**Additional file 1.**

**Additional file 2.**

**Additional file 3.**

## Footnotes

1 Protection seekers are foreigners staying in Germany on the basis of international law, humanitarian or political reasons and are registered in the Central Register of Foreigners (AZR) with the corresponding status under residence law. Terms such as refugees, asylum seekers or persons entitled to asylum are often used as synonyms for people who have fled, but in immigration and asylum law they only describe a specific subset of those seeking protection [2] The term “refugee” is used synonymously for protection seeker in this paper.

2 Cultural sensitivity as it is used here does refer to a static understanding of culture. It draws back to the need of knowledge about norms, values and attitudes of specific cultures while dismissing heterogeneity and transitions within and between cultures and individuals [46].
