# Supplementary material for: Good practice in reaching and treating refugees in addiction care in Germany – a Delphi study
Source: BMC Public Health. 2024 Jan 2;24:30. doi: 10.1186/s12889-023-17446-1 (PMC10763166; doi:10.1186/s12889-023-17446-1)
Supplement: Supplementary file 1 — Additional file 1. [file 12889_2023_17446_MOESM1_ESM.docx]

**DELPHI PROCESS "STRATEGIES OF "GOOD PRACTICE" REGARDING REACHING AND CARING FOR REFUGEES AND ASYLUM SEEKERS THROUGH ADDICTION SERVICES".**

**FIRST ROUND OF DATA COLLECTION**

In this first round of the Delphi process, we would like to ask you as experts about the strategies of "good practice" that you are aware of with regard to reaching and caring for refugees and asylum seekers through addiction support

- **Please name up to ten strategies of "good practice" in addiction support that lead to refugees and asylum seekers being reached and adequately cared for.**
- **Describe and justify the individual strategies in a few sentences.**

**Please consider refugees and asylum seekers who have immigrated to Germany since 2015 or later, of different ages and genders.**

**Strategy 1:**

**Strategy 2:**

**Strategy 3:**

**Strategy 4:**

**Strategy 5:**

**Strategy 6:**

**Strategy 7:**

**Strategy 8:**

**Strategy 9:**

**Strategy 10:**
