# Supplementary material for: Good practice in reaching and treating refugees in addiction care in Germany – a Delphi study
Source: BMC Public Health. 2024 Jan 2;24:30. doi: 10.1186/s12889-023-17446-1 (PMC10763166; doi:10.1186/s12889-023-17446-1)
Supplement: Supplementary file 3 — Additional file 3. [file 12889_2023_17446_MOESM3_ESM.docx]

**Consensus strategies**

**Care System**

| **1. Opening all existing addiction services to refugees.**  There are many established services and support structures in addiction services (e.g. Psychsocial support during substitution treatment (PSB), outpatient therapy, assisted forms living). These will be opened up for refugees nationwide in order to ensure seamless, continuous support, care and treatment close to home and in line with needs in all areas of addiction services (e.g. inpatient detoxification and withdrawal services, medical practices, outpatient addiction services). Change and further development processes are initiated at the individual and organisational level. | | | | |
| --- | --- | --- | --- | --- |
| **Not important** |  |  |  | **Very important** |
|  |  |  |  |  |
| **1** | **2** | **3** | **4** | **5** |
|  |  |  |  |  |

| **4. Overcoming municipally-based support structures**  Municipally-based service structures are flexibly overcome in order to be able to guarantee care. This takes into account the mobility of some refugees (e.g. their registered address differs from their place of residence) and creates continuity in care (despite e.g. frequent changes of accommodation). For this, supra-municipal cooperation is necessary. | | | | |
| --- | --- | --- | --- | --- |
| **Not important** |  |  |  | **Very important** |
|  |  |  |  |  |
| **1** | **2** | **3** | **4** | **5** |
|  |  |  |  |  |

| **6. Ensuring consistency of addiction support services**  Stable, sufficient and permanent funding of addiction support services is guaranteed in order to ensure consistency and clarity for both clients and staff. | | | | |
| --- | --- | --- | --- | --- |
| **Not important** |  |  |  | **Very important** |
|  |  |  |  |  |
| **1** | **2** | **3** | **4** | **5** |
|  |  |  |  |  |

**Framework conditions**

| **9. nationwide equal opportunities for refugees with regard to entitlement of benefits and right of use**  Unrestricted equal opportunities for refugees nationwide are guaranteed in order to enable utilisation of all health care and addiction support services. | | | | |
| --- | --- | --- | --- | --- |
| **Not important** |  |  |  | **Very important** |
|  |  |  |  |  |
| **1** | **2** | **3** | **4** | **5** |
|  |  |  |  |  |

| **10. Reduction of structural factors that facilitate or maintain addiction.**  Aspects of the living situation of refugees that facilitate addiction are taken seriously and reduced or eliminated, and social participation is made possible regardless of the legal residence status. Structural factors such as lengthy asylum procedures, unclear residence prospects, problems with family reunification, more difficult formal access to training and work, lack of recognition of foreign educational biographies and living in accommodation can influence substance use and addiction. | | | | |
| --- | --- | --- | --- | --- |
| **Not important** |  |  |  | **Very important** |
|  |  |  |  |  |
| **1** | **2** | **3** | **4** | **5** |
|  |  |  |  |  |

**Multilingualism**

| **11. Addressing people in their mother tongue as a gesture of welcome**  Addressing people in their mother tongue in all context (street work, (telephone) counselling) by means of language skills in the team or language mediators gives refugees the feeling of being welcome and understood. | | | | |
| --- | --- | --- | --- | --- |
| **Not important** |  |  |  | **Very important** |
|  |  |  |  |  |
| **1** | **2** | **3** | **4** | **5** |
|  |  |  |  |  |

| **12 Nationwide implementation of language mediation in addiction support facilities**  Working with language mediators will be implemented across the board in all areas involved and facilities of addiction support in order to ensure that all refugees can make use of regular care services and thus enable, for example, further referral in the context of addiction counselling or follow-up treatment after clinical withdrawal. Language mediators are used to minimise language barriers and misunderstandings. The language variety among refugees is diverse. Native-speaking staff cannot cover this diversity | | | | |
| --- | --- | --- | --- | --- |
| **Not important** |  |  |  | **Very important** |
|  |  |  |  |  |
| **1** | **2** | **3** | **4** | **5** |
|  |  |  |  |  |

| **13. Ensuring funding of language mediators**  Cost absorption of services for language mediators must be settled in institutions and by funding bodies. Costs are taken into account/calculated in the budget of institutions or financed by means of other additional funding. | | | | |
| --- | --- | --- | --- | --- |
| **Not important** |  |  |  | **Very important** |
|  |  |  |  |  |
| **1** | **2** | **3** | **4** | **5** |

| **14 Fast and low-threshold availability of language mediators**  Language mediators should be quickly and easily available for addiction support facilities, e.g. via a pool of language mediators - whether for face-to-face, telephone or video mediation. | | | | |
| --- | --- | --- | --- | --- |
| **Not important** |  |  |  | **Very important** |
|  |  |  |  |  |
| **1** | **2** | **3** | **4** | **5** |
|  |  |  |  |  |

| 1**5. Professionalism of the language mediators employed**  Language mediators are qualified for their work and act professionally. This includes, for example, correct translation without personal judgement. | | | | |
| --- | --- | --- | --- | --- |
| **Not important** |  |  |  | **Very important** |
|  |  |  |  |  |
| **1** | **2** | **3** | **4** | **5** |
|  |  |  |  |  |

| **16 Supervision for language mediators**  Offering supervision for language mediators contributes to their professionalism. | | | | |
| --- | --- | --- | --- | --- |
| **Not important** |  |  |  | **Very important** |
|  |  |  |  |  |
| **1** | **2** | **3** | **4** | **5** |
|  |  |  |  |  |

| **17 Multilingualism of documents of the facility process**  Documents from the facility's procedures, such as data protection declarations, treatment agreements and confidentiality agreements, are translated into- and used in different languages. | | | | |
| --- | --- | --- | --- | --- |
| **Not important** |  |  |  | **Very important** |
|  |  |  |  |  |
| **1** | **2** | **3** | **4** | **5** |
|  |  |  |  |  |

**Information and education**

|  | | | | |
| --- | --- | --- | --- | --- |
| **18. Provision of centrally designed, multilingual information on substances, substance use and addiction**  Refugees are informed about substances, substance use and the emergence and consequences of addiction multilingually, in easy-to-understand language and using multimedia. This can be done, for example, by means of centrally designed flyers, films, apps, social media, contributions in non-native German-language television programmes or broadcasts. Among other things, the focus is on establishing an understanding of addiction as a treatable health problem/illness. | | | | |
| **Not important** |  |  |  | **Very important** |
|  |  |  |  |  |
| **1** | **2** | **3** | **4** | **5** |
|  |  |  |  |  |

| **19. Passing on (bundled or centrally designed) multilingual information on addiction-related care services and framework conditions.**  Diverse addiction care services as well as their range of services and essential framework conditions (such as confidentiality, data protection, anonymity, addiction support as non-governmental) are publicised among refugees. This information can be bundled, e.g. prepared for all providers and facilities. It is important to make the information multilingual and easy to understand, for example by using pictograms and simple language, and to provide basic explanations of ubiquitous terms such as "counselling centre" or "therapy". | | | | |
| --- | --- | --- | --- | --- |
| **Not important** |  |  |  | **Very important** |
|  |  |  |  |  |
| **1** | **2** | **3** | **4** | **5** |
|  |  |  |  |  |

| **20. Outreach information work in the living environment of refugees.**  Prevention and education are undertaken where refugees are, e.g. in German/integration courses, self-help groups, neighbourhood centres, social media. In refugee accommodation (camps), for example, information events are offered by addiction support staff for all residents in their native languages (e.g. with the help of language mediators), easily understandable and interactive. | | | | |
| --- | --- | --- | --- | --- |
| **Not important** |  |  |  | **Very important** |
|  |  |  |  |  |
| **1** | **2** | **3** | **4** | **5** |
|  |  |  |  |  |

**Access**

| **21. Raising awareness of addiction issues among those involved in refugee assistance.**  Professionals and volunteers working with refugees are informed about substance use and addiction support services, e.g. through training courses. Insecurities in dealing with substances and addiction are thus reduced, substance use by refugees is recognised earlier and, if necessary, referral to addiction support services is offered.  Topics of the trainings conducted by addiction support staff are, for example, a possible approach to substance users, knowledge about substances, the course of addiction or the development of one's own attitude. | | | | |
| --- | --- | --- | --- | --- |
| **Not important** |  |  |  | **Very important** |
|  |  |  |  |  |
| **1** | **2** | **3** | **4** | **5** |
|  |  |  |  |  |

| **22. Use of key persons as door openers**  People who are in good contact with refugees (who use addictive substances) are considered key persons. They are actively used as multipliers to create access to the target group for addiction support services. Key persons can be parents, mothers, caregivers, stakeholders of a community or others. | | | | |
| --- | --- | --- | --- | --- |
| **Not important** |  |  |  | **Very important** |
|  |  |  |  |  |
| **1** | **2** | **3** | **4** | **5** |
|  |  |  |  |  |

| **24. Qualification of and work with "bridge builders”**  Bridge builders (so-called peers, buddies, health or integration facilitators, lay helpers, etc.) are qualified by addiction support services and actively integrated into the addiction support work - at least financially remunerated, at best as employees. Depending on the area in which they work, "bridge builders" bring similar experiences with regard to e.g. migration, sexual orientation, gender, substance use and have non-German mother tongue skills. The qualification and work with "bridge builders" includes continuous qualification, connection and supervision.  Bridge builders establish contact and trust with refugees and clarify (culturally conditioned) misunderstandings. They work in an outreach capacity, accompany and also provide support after referral or during an inpatient stay.  This work complements addiction support services, but does not replace them. | | | | |
| --- | --- | --- | --- | --- |
| **Not important** |  |  |  | **Very important** |
|  |  |  |  |  |
| **1** | **2** | **3** | **4** | **5** |
|  |  |  |  |  |

**service level**

| **25. Ensuring low-threshold access to addiction support services**  When designing services for refugees, access barriers (e.g. language, little prior knowledge, mistrust) are taken into account in order to make services low-threshold and in order to reach highly diverse people. Measures for this are, for example, the establishment of telephone services in different languages, open regular counselling services (also for relatives), services without prior appointment (e.g. in shelters) and a detailed presentation of the role and services of counselling centres at information events. | | | | |
| --- | --- | --- | --- | --- |
| **Not important** |  |  |  | **Very important** |
|  |  |  |  |  |
| **1** | **2** | **3** | **4** | **5** |
|  |  |  |  |  |

| **26. Emphasis on discretion and anonymity**  Discretion and anonymity are explained and emphasized to reduce shame, stigma and mistrust, especially when working with refugees (e.g. aspects of confidentiality, data protection against authorities), and are also taken into account when devising services (e.g. anonymous services in shelters or in the context of telephone counseling). | | | | |
| --- | --- | --- | --- | --- |
| **Not important** |  |  |  | **Very important** |
|  |  |  |  |  |
| **1** | **2** | **3** | **4** | **5** |
|  |  |  |  |  |

| **27. Ensuring participation and active involvement of people affected by addiction in the process of developing services and materials**  Refugees and persons in close contact with them (e.g. refugee assistance professionals) are involved in the development and conception of addiction support materials and services. | | | | |
| --- | --- | --- | --- | --- |
| **Not important** |  |  |  | **Very important** |
|  |  |  |  |  |
| **1** | **2** | **3** | **4** | **5** |
|  |  |  |  |  |

| **28. Participation of refugees in self-help activities**  Refugees are referred to existing self-help groups/initiatives and supported in setting up new groups/initiatives. This does not only refer to addiction-specific groups. | | | | |
| --- | --- | --- | --- | --- |
| **Not important** |  |  |  | **Very important** |
|  |  |  |  |  |
| **1** | **2** | **3** | **4** | **5** |
|  |  |  |  |  |

| **29. Early intervention for substance use among refugees**  Refugees are considered as potential patients in existing early intervention programs in the field of addiction support and are targeted as such. Cooperation with shelters, for example, is conceivable when it comes to preventive measures and early detection. | | | | |
| --- | --- | --- | --- | --- |
| **Not important** |  |  |  | **Very important** |
|  |  |  |  |  |
| **1** | **2** | **3** | **4** | **5** |
|  |  |  |  |  |

| **31. Outreach counseling in the immediate surroundings of refugees**  Counseling services regarding addiction and the (addiction) support system are offered in the (living) environment of refugees, e.g. in the form of regular open office hours in a shelter, in German/integration courses, self-help groups, neighborhood centers or social media. Language, anonymity and continuity are taken into account in the conception of these services in order to ensure low-threshold access. | | | | |
| --- | --- | --- | --- | --- |
| **Not important** |  |  |  | **Very important** |
|  |  |  |  |  |
| **1** | **2** | **3** | **4** | **5** |
|  |  |  |  |  |

| **32. Outreach work in places where drugs are consumed**  Outreach work (distribution of consumables, basic medical care, etc.) in places where drugs are used explicitly establishes contact with refugee users. | | | | |
| --- | --- | --- | --- | --- |
| **Not important** |  |  |  | **Very important** |
|  |  |  |  |  |
| **1** | **2** | **3** | **4** | **5** |
|  |  |  |  |  |

| **33. Outreach work to build up relationships**  In order to overcome inhibitions, create a climate of trust and build up relationships, addiction support workers are regularly present in the (living) environment of refugees. This can be implemented, for instance, within the context of regular “tea hours”/information events or by taking part in activities within e.g. refugee accommodations. Conversations are centered on topics relevant to the person and not on the care mandate of addiction support services. | | | | |
| --- | --- | --- | --- | --- |
| **Not important** |  |  |  | **Very important** |
|  |  |  |  |  |
| **1** | **2** | **3** | **4** | **5** |
|  |  |  |  |  |

| **34. Regularity and durability in the relational work with refugees.**  In order to create a trustful relationship, it is necessary to work with clients on a long-term and regular basis. Time is invested in the careful building of trust. Clients have fixed contact persons, e.g. the same staff member, the same language mediator. The focus is on long-term support and regular contact. | | | | |
| --- | --- | --- | --- | --- |
| **Not important** |  |  |  | **Very important** |
|  |  |  |  |  |
| **1** | **2** | **3** | **4** | **5** |
|  |  |  |  |  |

| **35. Accompanying clients**  If necessary, refugees are accompanied to appointments relevant to their addiction (e.g. authorities, day-structuring services, counseling centers, clinics). Accompaniment by a familiar professional often stabilizes the situation and facilitates the connection to high-threshold services (e.g. substitution). | | | | |
| --- | --- | --- | --- | --- |
| **Not important** |  |  |  | **Very important** |
|  |  |  |  |  |
| **1** | **2** | **3** | **4** | **5** |
|  |  |  |  |  |

**Employee level**

| **36. Trainings for addiction support professionals that addresses the living situation of refugees**  Addiction support professionals receive specialized training that addresses the complex socio-political conditions and living situations of refugees. The focus is on aspects of asylum and residence law, family reunification, regulations on coverage of costs and the responsibilities of cost bearers, refugee assistance services, employment opportunities, etc. **Addiction support professionals** are informed and made aware of these issues, but they do not take over tasks from other areas of work (such as (asylum) legal counseling centers). | | | | |
| --- | --- | --- | --- | --- |
| **Not important** |  |  |  | **Very important** |
|  |  |  |  |  |
| **1** | **2** | **3** | **4** | **5** |
|  |  |  |  |  |

| **38 Promoting diversity in teams**  In teams of addiction support facilities, professionals with a migration history and/or non-German (native) language skills are employed and included. The aim is not only to partially overcome language barriers, but also to change the discourse in facilities. In order to promote young professionals, the addiction support services make their field of work attractive to non-German mother-tongue students. | | | | |
| --- | --- | --- | --- | --- |
| **Not important** |  |  |  | **Very important** |
|  |  |  |  |  |
| **1** | **2** | **3** | **4** | **5** |
|  |  |  |  |  |

**Employee-attitudes**

| **39. Understanding and acceptance of substance use as a coping strategy**  Addiction problems can be a consequence of derailed self-medication of e.g. trauma sequelae (post-traumatic stress disorder). An understanding and accepting attitude towards individual substance use, the recognition of it as a coping strategy as well as a symptom and regulation of distress, is essential. Alternative coping strategies and regulatory mechanisms are worked out together within the framework of realistic possibilities of acting and caring for refugees. | | | | |
| --- | --- | --- | --- | --- |
| **Not important** |  |  |  | **Very important** |
|  |  |  |  |  |
| **1** | **2** | **3** | **4** | **5** |
|  |  |  |  |  |

| **40 Adopting an appreciative, living environment-oriented attitude**  Services and professionals do not focus homogenously on the history of flight or "being a refugee", but rather take into account the individual, heterogeneous living environments, backgrounds and biographies of refugee clients. Appreciation and resource orientation play an important role in this. The assistance is guided by the current needs, motivations and interests of the individual seeking help. | | | | |
| --- | --- | --- | --- | --- |
| **Not important** |  |  |  | **Very important** |
|  |  |  |  |  |
| **1** | **2** | **3** | **4** | **5** |
|  |  |  |  |  |

| **41. Cross- and transcultural competences in attitude and reflection**  Actors in addiction support have cross- and transcultural competence in the sense of a non-discriminatory attitude and self-reflection. Refugees are met with an open, curious and questioning attitude at eye level instead of with prejudice and judgement. The focus is not on the "culture" of the other person as conceived by the nation state or cultural circles, but on one's own attitude and ability to reflect.  Such an attitude is continuously challenged through training, supervision and self-reflection. This includes e.g.   - a reflection on one's own, often Eurocentric understanding of health and illness - a confrontation with discrimination, exclusion and racism as an expression of socially unequal positions and power relations as well as their multi-layered entanglements. | | | | |
| --- | --- | --- | --- | --- |
| **Not important** |  |  |  | **Very important** |
|  |  |  |  |  |
| **1** | **2** | **3** | **4** | **5** |
|  |  |  |  |  |

|  | | | | |
| --- | --- | --- | --- | --- |
| **42. Adopting a gender-sensitive attitude.**  Gender is not disregarded as an important social category, but is considered as part of the individual life situation. A gender-sensitive attitude can lead, for example, to the possibility of a gender-homogeneous group to discuss taboo topics or to gender-heterogeneous teams of professionals to meet the needs of clients. | | | | |
| **Not important** |  |  |  | **Very important** |
|  |  |  |  |  |
| **1** | **2** | **3** | **4** | **5** |
|  |  |  |  |  |

| **43 Coping mechanisms and setting boundaries as a competence of professionals**  When working with refugees because of e.g. insufficient German skills or because of their uncertain residence status, work can be less successful. Professionals in addiction support are in a position to distance themselves and to endure feelings of powerlessness and helplessness that arise. | | | | |
| --- | --- | --- | --- | --- |
| **Not important** |  |  |  | **Very important** |
|  |  |  |  |  |
| **1** | **2** | **3** | **4** | **5** |
|  |  |  |  |  |

**Networking**

| **44. Networking of all stakeholders involved in the care of drug users**  All stakeholders involved in the care of drug-using refugees exchange information regularly, support each other and work together synergistically. This means that not every agency has to cover everything (e.g. languages, group offers). | | | | |
| --- | --- | --- | --- | --- |
| **Not important** |  |  |  | **Very important** |
|  |  |  |  |  |
| **1** | **2** | **3** | **4** | **5** |
|  |  |  |  |  |

| **45. Multidisciplinary networking beyond addiction support services**.  Multidisciplinary networking beyond addiction support services (e.g. migration counselling, family counselling, help for the homeless, health care) is established. This aims at:   - Public relations work and publicising services from various fields of work in order to ensure that referrals can be made in line with needs - continuous public relations work in local/regional committees - an interdisciplinary exchange of expertise (e.g. Aylbewerberleistungsgesetz (Asylum Seekers' Benefits Act), accessibility of language mediators, early detection of risk behaviour, confidentiality) - Relationship work at the level of the agency as well as among professionals | | | | |
| --- | --- | --- | --- | --- |
| **Not important** |  |  |  | **Very important** |
|  |  |  |  |  |
| **1** | **2** | **3** | **4** | **5** |
|  |  |  |  |  |

| **47. Considering networking financially and conceptually**  Personnel and financial resources are applied for, made available and used for networking. In addition, a conceptual discussion about the objectives of networking takes place in advance. | | | | |
| --- | --- | --- | --- | --- |
| **Not important** |  |  |  | **Very important** |
|  |  |  |  |  |
| **1** | **2** | **3** | **4** | **5** |
|  |  |  |  |  |

| **48. Establishment of in-depth inter-institutional cooperation**  Inter-institutional cooperation, especially between addiction and refugee services, is developed and represents a deepening of networking. The focus is on joint conceptualisation and development of services and materials as well as on case-specific cooperation in the form of action guidelines and case conferences. A cooperation agreement is an important basis for clarifying responsibilities and procedures. | | | | |
| --- | --- | --- | --- | --- |
| **Not important** |  |  |  | **Very important** |
|  |  |  |  |  |
| **1** | **2** | **3** | **4** | **5** |
|  |  |  |  |  |

**Strategies without consensus**

| **2. Creation of specific services for refugees**.  New specialised services and facilities (inpatient and outpatient) for refugees are created in order to meet their cultural, linguistic and trauma-specific needs. | | | | |
| --- | --- | --- | --- | --- |
| **Not important** |  |  |  | **Very important** |
|  |  |  |  |  |
| **1** | **2** | **3** | **4** | **5** |
|  |  |  |  |  |

| **3. Establishment of services for individual subgroups of refugees.**  Services for individual subgroups of refugees (e.g. women, young people, languages) are established. These address specific needs. | | | | |
| --- | --- | --- | --- | --- |
| **Not important** |  |  |  | **Very important** |
|  |  |  |  |  |
| **1** | **2** | **3** | **4** | **5** |
|  |  |  |  |  |

| **5.Enabling flexible project development and implementation.**  During the implementation phase of projects, it is possible to develop them further and adapt them to unforeseen needs. | | | | |
| --- | --- | --- | --- | --- |
| **Not important** |  |  |  | **Very important** |
|  |  |  |  |  |
| **1** | **2** | **3** | **4** | **5** |
|  |  |  |  |  |

| **7. Management of requirements planning by policy-makers (federal government, state governments and municipalities)**  Federal states and municipalities should manage demand planning in close contact with the federal government, especially with regard to the expected number of refugees and their respective countries of origin. In this way, addiction support facilities are prepared for social changes and can react quickly to their needs. | | | | |
| --- | --- | --- | --- | --- |
| **Not important** |  |  |  | **Very important** |
|  |  |  |  |  |
| **1** | **2** | **3** | **4** | **5** |
|  |  |  |  |  |

| **8. Integration of science and practice**  Scientific findings should be incorporated into the practical work of addiction support. If possible, interventions are based on studies or scientifically sound guidelines. New approaches in addiction support practice are in turn scientifically reviewed with regard to evidence. | | | | |
| --- | --- | --- | --- | --- |
| **Not important** |  |  |  | **Very important** |
|  |  |  |  |  |
| **1** | **2** | **3** | **4** | **5** |
|  |  |  |  |  |

| **23. Working with relatives**  In groups that are not specific to addiction (e.g. parents' or women's groups), information is provided on topics such as drug use, addiction and the addiction support system. Relatives can share the acquired knowledge with their families. | | | | |
| --- | --- | --- | --- | --- |
| **Not important** |  |  |  | **Very important** |
|  |  |  |  |  |
| **1** | **2** | **3** | **4** | **5** |
|  |  |  |  |  |

| **30. Creating and maintaining a welcoming environment across all services.**  All addiction support services create and maintain a welcoming ambiance and a confidential atmosphere. Rooms are designed accordingly. Drinks and, if necessary, food are offered (e.g. at workshops). | | | | |
| --- | --- | --- | --- | --- |
| **Not important** |  |  |  | **Very important** |
|  |  |  |  |  |
| **1** | **2** | **3** | **4** | **5** |
|  |  |  |  |  |

| **37. Cultural sensitivity of professionals**  Addiction support professionals and professionals in other involved fields act in a culturally sensitive manner when dealing with refugees. The prerequisite for this is knowledge about the countries of origin and cultural backgrounds (politics, society and culture), cultural characteristics, religions, value systems and world views of refugees, as well as culture-specific characteristics of addiction and mental illness. Intercultural conflicts are also known (e.g. with regard to female professionals and male clients, hostile groups such as Sunni/Alevi/Shiite Muslims vs. Yezidi Kurds, etc.). | | | | |
| --- | --- | --- | --- | --- |
| **Not important** |  |  |  | **Very important** |
|  |  |  |  |  |
| **1** | **2** | **3** | **4** | **5** |
|  |  |  |  |  |

| **46. Networking with civil society stakeholders**  Networking with civil society stakeholders (e.g. churches, sports clubs, language schools, initiatives, migrant self-organisations) is established. This aims at:   - passing on information on substance use and addiction as well as services to refugees who are represented there (e.g. discussing the topic of health/addiction in German courses) - Knowledge of the services offered by civil society stakeholders to refer clients of addiction support services (e.g. leisure activities as an alternative strategy to consumption) to these services in a way that meets their needs. | | | | |
| --- | --- | --- | --- | --- |
| **Not important** |  |  |  | **Very important** |
|  |  |  |  |  |
| **1** | **2** | **3** | **4** | **5** |
|  |  |  |  |  |
